# Chronic Airways Assessment Test: psychometric properties in patients with asthma and/or COPD

**DOI:** 10.1186/s12931-023-02394-6

**Published:** 2023-04-08

**Authors:** Erin L. Tomaszewski, Mark J. Atkinson, Christer Janson, Niklas Karlsson, Barry Make, David Price, Helen K. Reddel, Claus F. Vogelmeier, Hana Müllerová, Paul W. Jones, NOVELTY Scientific Community;, Ricardo del Olmo, Gary Anderson, Helen Reddel, Marcelo Rabahi, Andrew McIvor, Mohsen Sadatsafavi, Ulla Weinreich, Pierre-Régis Burgel, Gilles Devouassoux, Alberto Papi, Hiromasa Inoue, Adrian Rendon, Maarten van den Berge, Richard Beasley, Alvar Agustí García-Navarro, Rosa Faner, José Olaguibel Rivera, Christer Janson, Magdalena Bilińska-Izydorczyk, Malin Fagerås, Titti Fihn-Wikander, Stefan Franzén, Christina Keen, Kristoffer Ostridge, James Chalmers, Timothy Harrison, Ian Pavord, David Price, Adnan Azim, Laura Belton, Francois-Xavier Blé, Clement Erhard, Kerry Gairy, Rod Hughes, Glenda Lassi, Hana Müllerová, Eleni Rapsomaniki, Ian Christopher Scott, Bradley Chipps, Barry Make, Stephanie Christenson, Erin Tomaszewski, NOVELTY study investigators, Ricardo del Olmo, Gabriel Benhabib, Xavier Bocca Ruiz, Raul Eduardo Lisanti, Gustavo Marino, Walter Mattarucco, Juan Nogueira, Maria Parody, Pablo Pascale, Pablo Rodriguez, Damian Silva, Graciela Svetliza, Carlos F. Victorio, Roxana Willigs Rolon, Anahi Yañez, Helen Reddel, Stuart Baines, Simon Bowler, Peter Bremner, Sheetal Bull, Patrick Carroll, Mariam Chaalan, Claude Farah, Gary Hammerschlag, Kerry Hancock, Zinta Harrington, Gregory Katsoulotos, Joshua Kim, David Langton, Donald Lee, Matthew Peters, Lakshman Prassad, Dimitar Sajkov, Francis Santiago, Frederick Graham Simpson, Sze Tai, Paul Thomas, Peter Wark, Marcelo Rabahi, José Eduardo Delfini Cançado, Thúlio Cunha, Marina Lima, Alexandre Pinto Cardoso, J. Mark FitzGerald, Andrew McIvor, Syed Anees, John Bertley, Alan Bell, Amarjit Cheema, Guy Chouinard, Michael Csanadi, Anil Dhar, Ripple Dhillon, David Kanawaty, Allan Kelly, William Killorn, Daniel Landry, Robert Luton, Piushkumar Mandhane, Bonavuth Pek, Robert Petrella, Daniel Stollery, Chen Wang, Meihua Chen, Yan Chen, Wei Gu, Kim Ming Christopher Hui, Manxiang Li, Shiyue Li, Ma Lijun, Guangyue Qin, Weidong Song, Wei Tan, Yijun Tang, Tan Wang, Fuqiang Wen, Feng Wu, PingChao Xiang, Zuke Xiao, Shengdao Xiong, Jinghua Yang, Jingping Yang, Caiqing Zhang, Min Zhang, Ping Zhang, Wei Zhang, Xiaohe Zheng, Dan Zhu, Carlos Matiz Bueno, Fabio Bolivar Grimaldos, Alejandra Cañas Arboleda, Dora Molina de Salazar, Ulla Weinreich, Elisabeth Bendstrup, Ole Hilberg, Carsten Kjellerup, Pierre-Régis Burgel, Gilles Devouassoux, Chantal Raherison, Philippe Bonniaud, Olivier Brun, Christos Chouaid, Francis Couturaud, Jacques de Blic, Didier Debieuvre, Dominique Delsart, Axelle Demaegdt, Pascal Demoly, Antoine Deschildre, Carole Egron, Lionel Falchero, François Goupil, Romain Kessler, Pascal Le Roux, Pascal Mabire, Guillaume Mahay, Stéphanie Martinez, Boris Melloni, Laurent Moreau, Emilie Riviere, Pauline Roux-Claudé, Michel Soulier, Guillaume Vignal, Azzedine Yaici, Robert Bals, Sven Philip Aries, Ekkehard Beck, Andreas Deimling, Jan Feimer, Vera Grimm-Sachs, Gesine Growth, Felix Herth, Gerhard Hoheisel, Frank Kanniess, Thomas Lienert, Silke Mronga, Jörg Reinhardt, Christian Schlenska, Christoph Stolpe, Ishak Teber, Hartmut Timmermann, Thomas Ulrich, Peter Velling, Sabina Wehgartner-Winkler, Juergen Welling, Ernst-Joachim Winkelmann, Alberto Papi, Carlo Barbetta, Fulvio Braido, Vittorio Cardaci, Enrico Maria Clini, Maria Teresa Costantino, Giuseppina Cuttitta, Mario di Gioacchino, Alessandro Fois, Maria Pia Foschino-Barbaro, Enrico Gammeri, Riccardo Inchingolo, Federico Lavorini, Antonio Molino, Eleonora Nucera, Vincenzo Patella, Alberto Pesci, Fabio Ricciardolo, Paola Rogliani, Riccardo Sarzani, Carlo Vancheri, Rigoletta Vincenti, Hiromasa Inoue, Takeo Endo, Masaki Fujita, Yu Hara, Takahiko Horiguchi, Keita Hosoi, Yumiko Ide, Minehiko Inomata, Koji Inoue, Sumito Inoue, Motokazu Kato, Masayuki Kawasaki, Tomotaka Kawayama, Toshiyuki Kita, Kanako Kobayashi, Hiroshi Koto, Koichi Nishi, Junpei Saito, Yasuo Shimizu, Toshihiro Shirai, Naruhiko Sugihara, Ken-ichi Takahashi, Hiroyuki Tashimo, Keisuke Tomii, Takashi Yamada, Masaru Yanai, Adrian Rendon, Ruth Cerino Javier, Alfredo Domínguez Peregrina, Marco Fernández Corzo, Efraín Montano Gonzalez, Alejandra Ramírez-Venegas, Maarten van den Berge, Willem Boersma, R. S. Djamin, Michiel Eijsvogel, Frits Franssen, Martijn Goosens, Lidwien Graat-Verboom, Johannes in’t Veen, Rob Janssen, Kim Kuppens, Mario van de Ven, Per Bakke, Ole Petter Brunstad, Gunnar Einvik, Kristian Jong Høines, Alamdar Khusrawi, Torbjorn Oien, Ho Joo Yoon, Yoon-Seok Chang, Young Joo Cho, Yong Il Hwang, Woo Jin Kim, Young-Il Koh, Byung-Jae Lee, Kwan-Ho Lee, Sang-Pyo Lee, Yong Chul Lee, Seong Yong Lim, Kyung Hun Min, Yeon-Mok Oh, Choon-Sik Park, Hae-Sim Park, Heung-Woo Park, Chin Kook Rhee, Hyoung-Kyu Yoon, Alvar Agustí García-Navarro, José Olaguibel Rivera, Rubén Andújar, Laura Anoro, María Buendía García, Paloma Campo Mozo, Sergio Campos, Francisco Casas Maldonado, Manuel Castilla Martínez, Carolina Cisneros Serrano, Lorena Comeche Casanova, Dolores Corbacho, Felix Del Campo Matías, Jose Echave-Sustaeta, Gloria Francisco Corral, Pedro Gamboa Setién, Marta García Clemente, Ignacio García Núñez, Jose García Robaina, Mercedes García Salmones, Jose Maria Marín Trigo, Marta Nuñez Fernandez, Sara Nuñez Palomo, Luis Pérez de Llano, Ana Pueyo Bastida, Ana Rañó, José Rodríguez González-Moro, Albert Roger Reig, José Velasco Garrido, Christer Janson, Dan Curiac, Cornelia Lif-Tiberg, Anders Luts, Lennart Råhlen, Stefan Rustscheff, Timothy Harrison, Frances Adams, Drew Bradman, Emma Broughton, John Cosgrove, Patrick Flood-Page, Elizabeth Fuller, David Hartley, Keith Hattotuwa, Gareth Jones, Keir Lewis, Lorcan McGarvey, Alyn Morice, Preeti Pandya, Manish Patel, Kay Roy, Ramamurthy Sathyamurthy, Swaminathan Thiagarajan, Alice Turner, Jorgen Vestbo, Wisia Wedzicha, Tom Wilkinson, Pete Wilson, Bradley Chipps, Lo’Ay Al-Asadi, James Anholm, Francis Averill, Sandeep Bansal, Alan Baptist, Colin Campbell, Michael A. Campos, Gretchen Crook, Samuel DeLeon, Alain Eid, Ellen Epstein, Stephen Fritz, Hoadley Harris, Mitzie Hewitt, Fernando Holguin, Golda Hudes, Richard Jackson, Alan Kaufman, David Kaufman, Ari Klapholz, Harshavardhan Krishna, Daria Lee, Robert Lin, Diego Maselli-Caceres, Vinay Mehta, James N. Moy, Ugo Nwokoro, Purvi Parikh, Sudhir Parikh, Frank Perrino, James Ruhlmann, Catherine Sassoon, Russell A. Settipane, Daniel Sousa, Peruvemba Sriram, Richard Wachs

**Affiliations:** 1 BioPharmaceuticals Medical, AstraZeneca, 1 Medimmune Way, Gaithersburg, MD USA; 2 Evidera, Bethesda, MD USA; 3 Department of Medical Sciences: Respiratory, Allergy and Sleep Research, Uppsala University, Uppsala, Sweden; 4 BioPharmaceuticals Medical, AstraZeneca, Gothenburg, Sweden; 5 National Jewish Health and University of Colorado Denver, Denver, CO USA; 6 Observational and Pragmatic Research Institute, Singapore, Singapore; 7 Centre of Academic Primary Care, Division of Applied Health Sciences, University of Aberdeen, Aberdeen, UK; 8 The Woolcock Institute of Medical Research, The University of Sydney, Sydney, NSW Australia; 9 Department of Medicine, Pulmonary and Critical Care Medicine, German Center for Lung Research (DZL), University of Marburg, Marburg, Germany; 10 BioPharmaceuticals Medical, AstraZeneca, Cambridge, UK; 11 Global Respiratory Franchise, GlaxoSmithKline, Brentford, Middlesex UK

**Keywords:** Asthma, COPD, Patient-reported, Psychometrics, Chronic Airways Assessment Test, COPD Assessment Test

## Abstract

**Background:**

No short patient-reported outcome (PRO) instruments assess overall health status across different obstructive lung diseases. Thus, the wording of the introduction to the Chronic Obstructive Pulmonary Disease (COPD) Assessment Test (CAT) was modified to permit use in asthma and/or COPD. This tool is called the Chronic Airways Assessment Test (CAAT).

**Methods:**

The psychometric properties of the CAAT were evaluated using baseline data from the NOVELTY study (NCT02760329) in patients with physician-assigned asthma, asthma + COPD or COPD. Analyses included exploratory/confirmatory factor analyses, differential item functioning and analysis of construct validity. Responses to the CAAT and CAT were compared in patients with asthma + COPD and those with COPD.

**Results:**

CAAT items were internally consistent (Cronbach’s alpha: > 0.7) within each diagnostic group (n = 510). Models for structural and measurement invariance were strong. Tests of differential item functioning showed small differences between asthma and COPD in individual items, but these were not consistent in direction and had minimal overall impact on the total score. The CAAT and CAT were highly consistent when assessed in all NOVELTY patients who completed both (N = 277, Pearson’s correlation coefficient: 0.90). Like the CAT itself, CAAT scores correlated moderately (0.4–0.7) to strongly (> 0.7) with other PRO measures and weakly (< 0.4) with spirometry measures.

**Conclusions:**

CAAT scores appear to reflect the same health impairment across asthma and COPD, making the CAAT an appropriate PRO instrument for patients with asthma and/or COPD. Its brevity makes it suitable for use in clinical studies and routine clinical practice.

*Trial registration*: NCT02760329.

**Supplementary Information:**

The online version contains supplementary material available at 10.1186/s12931-023-02394-6.

## Plain language summary

Chronic Airways Assessment Test: a questionnaire adapted to assess overall health in asthma and/or COPD

**What is it about?**

Asthma and chronic obstructive pulmonary disease (COPD) can have a significant effect on a person’s health and wellbeing. For people with these conditions, most of the health questionnaires available for use in patient care are designed for one specific diagnosis – either asthma (e.g. Asthma Control Test) or COPD (e.g. COPD Assessment Test [CAT]). A small number of questionnaires are available for use in both asthma and COPD, but these either take too long to complete, or do not focus on the overall health of patients. Since the symptoms in asthma and COPD overlap, a health questionnaire is needed for use in both asthma and COPD that can be completed during a routine visit to a doctor. This questionnaire could also help with research into the impact of lung diseases in people who have an unclear diagnosis.

The CAT was changed so that people with asthma or COPD (or both) could use the same test. We named this new version the Chronic Airways Assessment Test (CAAT). Our goal was to determine whether CAAT scores meant the same level of health in people with asthma, COPD, or both conditions.

The CAAT has eight questions, each scored 0 to 5, and takes only a few minutes to complete. The first three questions ask about the person’s symptoms, including how often they cough and whether they have chest mucus or chest tightness. The remaining five questions ask how the person’s asthma or COPD (or both) affects their daily life. These questions ask about shortness of breath, difficulties doing activities at home, confidence in leaving their home, whether they sleep soundly and how much energy they have. We found that the CAAT performed similarly in people with asthma or COPD (or both). This indicates that the CAAT score means a similar thing for someone with asthma, COPD or both conditions.

**Why is it important?**

The CAAT is a quick and simple way for patients to share with their doctor how bad their symptoms are and how much they affect their daily life. The development of the CAAT means that only one questionnaire is needed to measure the effect of a person’s symptoms on their current level of health, whether they have asthma, COPD, or both. This will allow doctors to improve the level of care patients receive.

**Supplementary Information:**

The online version contains supplementary material available at 10.1186/s12931-023-02394-6.

## Introduction

Asthma and chronic obstructive pulmonary disease (COPD) can substantially impact patient health status [1, 2]. Capturing patient-reported outcomes (PROs) is a key method for assessing patients in routine clinical practice and understanding the effects of treatments in clinical trials; regulatory authorities around the world have issued guidance on the collection of such patient-reported data [3–5].

Several PRO instruments assessing symptoms, impacts and health status have been specifically developed for asthma or COPD [6, 7] and use disease-specific wording. Only two respiratory health status instruments are currently available for use in both asthma and COPD: the St George’s Respiratory Questionnaire (SGRQ) [8] and the Airways Questionnaire 20 (AQ20) [2, 9]. Both of these have features that limit their use in the routine clinical setting. The SGRQ takes around 10–15 min to complete [7], making it impractical for routine use. The AQ20, by contrast, only takes 2–3 min to complete, but it focuses primarily on impairment and not overall health status [2, 9].

Given the overlap in symptoms between asthma and COPD [10–12], there is the potential to create a standardised health status measure for use in both asthma and COPD that is practical for administration in routine clinical practice and includes items relevant to both conditions. Such a measure may also enable research into the impact of obstructive lung disease in populations where the specific diagnosis may be unclear.

The widely used COPD Assessment Test (CAT) [13–15], a health status measure for COPD, was modified to replace disease-specific terms with generic ‘pulmonary disease’ language. This modified version is called the Chronic Airways Assessment Test (CAAT).

The goal of this analysis was to examine the psychometric properties of the CAAT in patients with asthma and/or COPD using cross-sectional data from the NOVELTY study (a NOVEL observational longiTudinal studY; NCT02760329) [12, 16].

## Materials and methods

### Development of the CAAT

The CAT was modified, with permission, to replace the term ‘COPD’ with ‘chronic airways’ in the title and replace ‘COPD’ with ‘pulmonary disease’ in the introduction. All other features of the CAAT are identical to the CAT, including the items, response options and scoring algorithm [13]. The CAT is the copyright of GlaxoSmithKline; the CAAT will similarly be placed under copyright with the same permissions for personal use, for clinical practice and for clinical research as the CAT.

The CAAT takes about 2–3 min to complete, and comprises eight items relating to respiratory symptoms (items 1–3: relating to cough, chest phlegm and chest tightness) and functional impacts on wellbeing and daily life (items 4–8: relating to breathlessness, activity limitation at home, confidence leaving home, ability to sleep soundly and energy level) (Additional file 1: Fig S1).

As with the CAT [13], the CAAT total score (range: 0–40) is calculated as the sum of the eight individual items, with higher scores indicating a worse health status. To calculate the CAAT total score, patients must provide responses to at least six items; if one or two responses are missing, the scores for the missing items are set to the average of the individual’s non-missing item scores at the time of administration.

### Psychometric validation sample

The goal of this study was to evaluate the cross-sectional psychometric properties of the CAAT from baseline data in patients with physician-assigned asthma and/or COPD in the NOVELTY study using item response theory (IRT) modelling and differential item functioning (DIF).

The total sample was selected from NOVELTY patients who completed the CAAT, and was comprised of three randomly selected sub-samples of patients with physician-assigned asthma, asthma + COPD or COPD (Additional file 1: Fig S2). Simple random sampling provided a balanced representation of patient demographics and severity categories reflective of the NOVELTY study. A second analytic sample of NOVELTY patients was comprised of those with asthma + COPD or COPD who completed both the CAAT and CAT (Additional file 1: Fig S2).

Sample size was based on observations needed to adequately power key sub-group analyses. A conservative approach was taken; diagnostic group sample sizes (N = 510) were double those previously reported to be required for accurate assessment [17].

For IRT-based DIF analysis, a sample size of 100–200 for 10 items is appropriate [18]. To obtain severity-balanced samples of this size, three 10% random samples were taken from the asthma and COPD groups and then combined, with duplicates removed (Additional file 1: Fig S2). A fourth COPD sample was taken to ensure adequate representation of patients assessed as having very severe COPD. No patients with asthma were assessed as having very severe disease. Patients with asthma + COPD were not eligible for DIF analysis due to the need for discretely characterised individuals (i.e. asthma only or COPD only).

### Psychometric validation objectives and analysis

The broad psychometric objectives of this work were to evaluate the items and scales of the CAAT for: (1) internal consistency and structural validity; (2) item response characteristics and conceptual framework of the CAAT using IRT modelling and DIF; (3) discriminant/concurrent validity; and (4) to compare the CAAT and CAT in the same patients.

Objective 1: The internal consistency of CAAT items is a necessary characteristic of overall construct validity (i.e. respiratory health status). Cronbach’s alpha was used to indicate the level of consistency between CAAT items (an alpha > 0.7 represents adequate consistency and > 0.9 suggests redundant items) [19]. Exploratory factor analysis (EFA) was then used to assess whether the CAAT items measured the same concept, or factor. Unidimensionality and structural validity were evaluated, with these findings then informing subsequent confirmatory factor analysis (using Mplus v8.2) and evaluation of the invariance of measurement and structural characteristics across diagnostic groupings.

Objective 2: The boundary locations, discrimination and information functions of CAAT items were examined using IRT analysis. A two-parameter logistic graded response model [20] was fitted using STATA v16.1 to examine item response characteristics. DIF analysis was performed to determine if CAAT items performed in the same way in patients with asthma and COPD using ordinal logistic regression (DifDetect in STATA [21]). Differences in response were explored by testing each item for uniform DIF (the presence of a mean difference between groups) and non-uniform DIF (differences between individuals changing across the response severity range).

Differences in DIF magnitude between groups were evaluated in two ways: effect sizes calculated as Cohen’s d, which give a dimensionless measure of the size of differences, and mean boundary difference scores across all five response options to each item, which provide an estimate of differences expressed in CAAT units. Based on prior findings for the CAT [22], a mean difference of two units was assumed to be the minimum clinically important difference (MCID) for the CAAT total score. For the purposes of this analysis, the MCID was assumed to be distributed equally across items, resulting in a CAAT item-level MCID of 0.25.

Objective 3: To evaluate convergent and discriminant validity (ability of a tool to relate to similar measures, and not relate well to measures that reflect a different aspect of disease), Pearson’s correlations between the CAAT and other measures were examined. Correlation coefficients > 0.70 were regarded as strong; 0.4–0.7 moderate; and < 0.4 weak [23]. Analysis of covariance was used to examine the relationship between CAAT and SGRQ total scores.

Objective 4: To evaluate the agreement between the CAAT and CAT in all patients from NOVELTY who completed both instruments (N = 277), intraclass correlation coefficients were used. The CAAT and CAT were further compared using descriptive statistics and Bland–Altman plots.

### NOVELTY study population

Observations were obtained from NOVELTY, a global, prospective, 3-year observational study of patients with a physician-assigned or suspected diagnosis of asthma and/or COPD [12, 16]. The study design, patient population, and ethical committee and institutional review board compliance have been reported previously [12, 16]. To avoid the selection bias observed in regulatory studies [24], NOVELTY enrolment was stratified by physician-assigned diagnosis (asthma, both asthma and COPD [hereafter referred to as asthma + COPD] or COPD) and physician-assessed severity (mild, moderate or severe). No diagnostic or severity criteria were pre-specified when determining eligibility. For patients with asthma + COPD, physician-assessed severity was the higher of the separate severity classifications for asthma and COPD.

### Data collection

Patients completed questionnaires via the web or by telephone interviews. Consistency in mode of PRO administration (i.e. web or telephone) was encouraged. The PROs were administered in the same order each time up until 2 July 2019. Thereafter, PROs could be completed in any order.

At baseline, patients were administered the CAAT, SGRQ and EuroQol 5-dimensions 5-level visual analogue scale (EQ-5D-5L VAS). A subset of patients with asthma + COPD or COPD completed both the CAAT and CAT. Additionally, data for spirometry measures (post-bronchodilator forced expiratory volume in 1 s [FEV_1_], forced vital capacity [FVC] and FEV_1_/FVC ratio) were collected at the baseline visit.

## Results

### Patient demographics and clinical characteristics

The total sample consisted of 1530 observations (510 in each diagnostic group). In the total sample, the mean and standard deviation was 62.4 ± 13.3 for age, 15.6 ± 16.9 for years since diagnosis, 70.6 ± 24.1 for post-bronchodilator FEV_1_% predicted, and 15.9 ± 8.5 for CAAT score (Table 1). Compared to the asthma + COPD and COPD groups, the asthma group was on average younger, had a higher proportion of females, had a lower mean CAAT score and higher mean post-bronchodilator FEV_1_% predicted (Table 1). Mean scores for the CAAT and CAT were similar among patients with asthma + COPD or COPD who completed both questionnaires (Table 1 and Additional file 1: Table S1).

**Table 1:** Patient demographics and clinical assessments by physician-assigned diagnosis

Variable	Asthma (N = 510)	Asthma + COPD (N = 510)	COPD (N = 510)	Total sample (N = 1530)
Age, years, mean (SD)	54.6 (15.7)	65.2 (9.9)	67.3 (9.6)	62.4 (13.3)
Female, n (%)	328 (64.3)	240 (47.1)	203 (39.8)	771 (50.4)
Ethnicity, n (%)				
African American	15 (2.9)	17 (3.3)	15 (2.9)	47 (3.1)
Caucasian	367 (72.0)	398 (78.0)	441 (86.5)	1206 (78.8)
North-east Asian	82 (16.1)	76 (14.9)	35 (6.9)	193 (12.6)
South-east Asian	9 (1.8)	5 (1.0)	1 (0.2)	15 (1.0)
Other	37 (7.3)	14 (2.7)	18 (3.5)	69 (4.5)
Time since diagnosis, years				
Patients with data, n	466	465	465	1396
Mean (SD)	17.8 (16.1)	21.0 (20.5)	8.1 (9.1)	15.6 (16.9)
Physician-assessed severity,^a^ n (%)				
Mild	181 (35.5)	70 (13.7)	151 (29.6)	402 (26.3)
Moderate	190 (37.3)	233 (45.7)	142 (27.8)	565 (36.9)
Severe	139 (27.3)	207 (40.6)	217 (42.5)	563 (36.8)
Post-bronchodilator FEV_1_ (% predicted)				
Patients with data, n	400	433	420	1253
Mean (SD)	85.6 (20.5)	66.7 (20.8)	60.4 (23.6)	70.6 (24.1)
Post-bronchodilator FEV_1_/FVC (% predicted)				
Patients with data, n	400	431	419	1250
Mean (SD)	91.8 (13.9)	75.1 (18.1)	71.5 (20.8)	79.2 (19.9)
SGRQ total score^b^				
Patients with data, n	500	502	501	1503
Mean (SD)	29.6 (20.2)	40.6 (22.2)	41.7 (21.2)	37.3 (21.9)
EQ-5D-5L VAS score^b^				
Patients with data, n	434	451	450	1335
Mean (SD)	74.4 (17.5)	67.2 (20.0)	64.8 (20.2)	68.7 (19.7)
CAAT total score^c^				
Patients with data, n	510	510	510	1530
Mean (SD)	13.7 (8.2)	17.2 (8.6)	16.9 (8.2)	15.9 (8.5)
CAT total score^c^				
Patients with data, n	NA	37	46	83
Mean (SD)	NA	15.1 (9.0)	16.5 (9.2)	15.9 (9.1)
**All NOVELTY patients who completed both the CAAT and CAT (N = 277)**^﻿d^				
CAAT total score^c^				
Patients with data, n	NA	66	211	277
Mean (SD)	NA	16.6 (9.5)	16.0 (8.8)	16.2 (8.9)
CAT total score^c^				
Patients with data, n	NA	66	211	277
Mean (SD)	NA	15.5 (9.8)	15.0 (8.6)	15.1 (8.9)

Patients with missing data were not included

*CAAT* Chronic Airways Assessment Test, *CAT* COPD Assessment Test, *COPD* chronic obstructive pulmonary disease, *EQ-5D-5L VAS* EuroQol 5-dimensions 5-level visual analogue scale, *FEV*_*1*_ forced expiratory volume in 1 s, *N* total number of patients in the sample, *n* number of patients with non-missing data, *NA* not applicable, *SD* standard deviation, *SGRQ* St George’s Respiratory Questionnaire

^a^For patients with asthma + COPD, severity was allocated as the higher of the two severity categories assigned by the physician for their asthma and their COPD; ^b^Range: 0–100; ^c^Range: 0–40; ^d^Data for all NOVELTY patients with asthma + COPD or COPD who completed the CAAT and CAT, including those not represented in the N = 1530 total sample

### Internal consistency and structural validity

Internal consistency was adequate (Cronbach’s alpha for asthma: 0.87; asthma + COPD: 0.86; COPD: 0.84; total sample: 0.86), indicating the CAAT assesses the same general construct as the CAT in each diagnostic group. Initial exploratory factor analyses indicated that items clustered into two correlated groups: items 1–3 pertaining to symptoms, and items 4–8 pertaining to functional impact. However, confirmatory factor analysis demonstrated very good fit of a single hierarchical factor for total CAAT score across diagnostic groups (Additional file 1: Table S2).

### Item response characteristics and conceptual framework

#### Item response theory

In the total sample, CAAT items had a good overall IRT model fit except item 6 (confidence leaving home). Item response boundary locations were monotonic and in the expected order. Discrimination between response options for individual items ranged from 1.2 to 2.9 across the health status continuum (theta). Symptom-related items (items 1–3) had broad coverage but were less informative (i.e. lacked precision) vs. the other items (Additional file 1: Fig S3). By comparison, functional impact-related items (items 4–8) provided more information but had a narrower range (Additional file 1: Fig S3). Across the total sample, test information coverage lay between theta values of −2.0 and 3.1.

#### Differential item function

Sampling resulted in 127 patients with asthma and 161 patients with COPD once duplicates were removed. Four items showed uniform DIF (p < 0.005; Table 2); none showed non-uniform DIF (p = 0.18–0.98). There was no consistent mean boundary difference in CAAT units between asthma and COPD (Table 2); patients with asthma scored lower (indicated by the negative sign) in five items and patients with COPD scored lower in three items. Assuming an item-level MCID of 0.25 (see Methods), this threshold was exceeded in five items. On average, the asthma group scored slightly lower, largely due to items 4 and 5; the mean difference was −0.19 (standard deviation 0.47; p > 0.1). This translates into 1.54 CAAT units, which is 3.9% of the scaling range of 0–40 and below the assumed two-unit CAAT MCID.

**Table 2:** Mean boundary difference and p values for uniform DIF between patients with asthma and COPD^a^

Item	Description	Mean boundary difference (Asthma–COPD)	p value for uniform DIF
1	Cough	0.559	0.003579
2	Phlegm	–0.012	0.282105
3	Chest tightness	0.072	0.126434
4	Breathlessness	–0.824	0.000009
5	Home activity	–0.724	0.000002
6	Confidence	–0.448	0.12692
7	Sleep	0.162	0.000737
8	Energy	–0.324	0.516946

Mean boundary difference for each CAAT item was calculated as the mean CAAT item score for patients with asthma subtracted by the mean CAAT item score for patients with COPD. A negative value indicates a lower response in patients with asthma. Responses to individual CAAT items ranged from 0 to 5

*CAAT* Chronic Airways Assessment Test, *COPD* chronic obstructive pulmonary disease, *DIF* differential item functioning

^a^None of the CAAT items showed significant non-uniform DIF

**Fig. 1:**
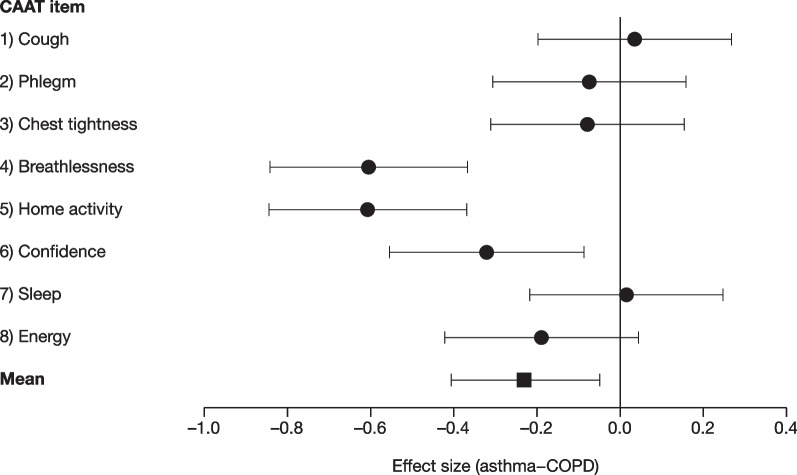
Mean boundary threshold difference between asthma and COPD for CAAT items by effect size units. Each CAAT item was scored between 0 and 5. Effect size units were calculated using Cohen’s d. A negative value indicates a lower response in patients with asthma. The overall mean was calculated as the standardised mean difference from a meta-analysis using a random effects model. Error bars represent 95% confidence intervals. *CAAT* Chronic Airways Assessment Test, *COPD* chronic obstructive pulmonary disease

When measured by effect size, three items (4, 5 and 6) showed a significantly lower response in patients with asthma (Fig. 1). A meta-analysis of all items demonstrated a significantly lower response overall in the asthma group (p = 0.013), but the difference was small (Cohen’s d =  −0.23).

### Convergent and divergent validity

**Fig. 2:**
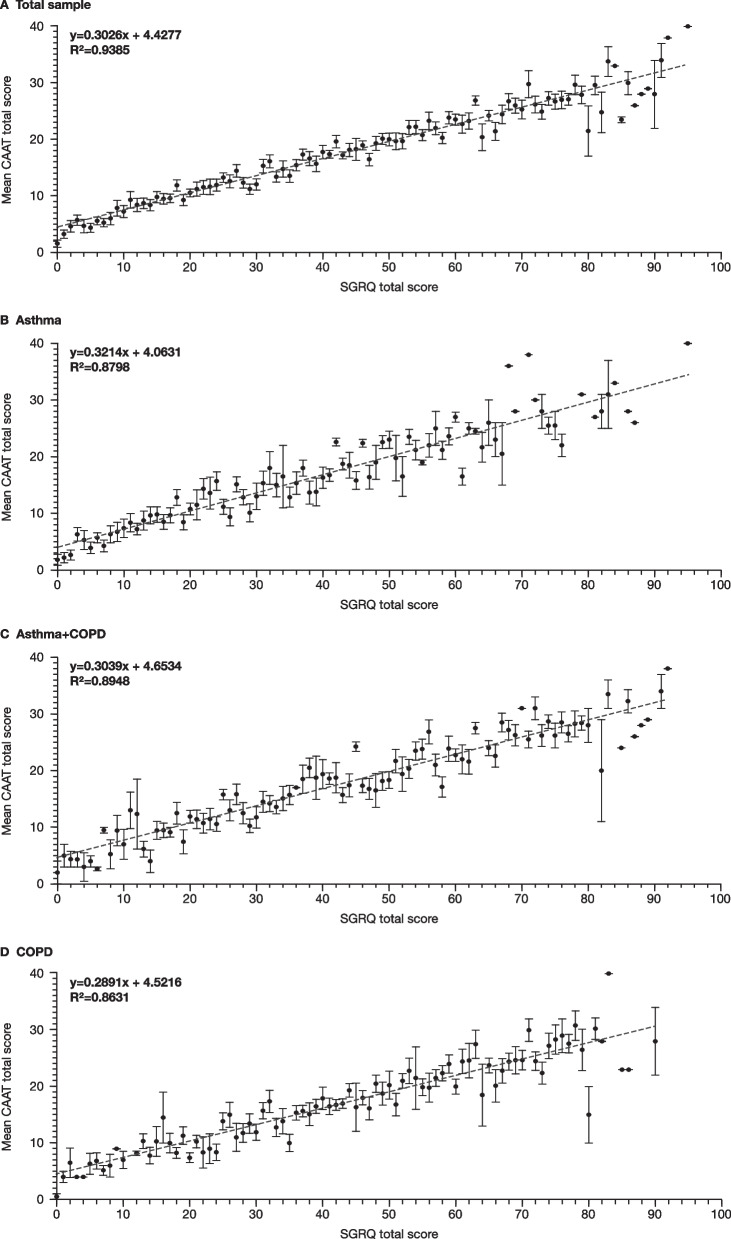
Linearity between CAAT and SGRQ total scores for the total sample and each diagnostic group. Error bars represent standard errors; data points with no error bars are representative of one patient with that SGRQ score. *CAAT* Chronic Airways Assessment Test, *COPD* chronic obstructive pulmonary disease, *SGRQ* St George’s Respiratory Questionnaire

Results showed consistently high correlation as reflected by R^2^ > 0.86 between the SGRQ and CAAT across diagnostic groups (Fig. 2; individual patient scores shown in Additional file 1: Fig S4). Analysis of covariance showed no significant difference in the regression slopes between asthma and COPD (p = 0.46). There was a significant intercept in all groups (i.e. when the SGRQ score was 0, the CAAT score was > 0 [≈ 5 units, p < 0.0001]), but there was no significant difference in intercept between asthma and COPD (p = 0.078).

The CAAT also correlated strongly with the CAT and moderately with the EQ-5D-5L VAS; weaker correlations were observed for spirometry measures (Table 3; Additional file 1: Table S3).

**Table 3:** Pearson’s correlations between CAAT score and patient-reported outcomes or clinical assessments

Variable^a^	Asthma (N = 510)	Asthma + COPD (N = 510)	COPD (N = 510)	Total sample (N = 1530)
SGRQ total score				
Patients with data available, n	500	502	501	1503
Pearson’s correlation coefficient	0.79***	0.81***	0.76***	0.79***
EQ-5D-5L VAS				
Patients with data available, n	434	451	450	1335
Pearson’s correlation coefficient	−0.53***	−0.56***	−0.57***	−0.57***
CAT total score^b^				
Patients with data available, n	NA	66	211	277
Pearson’s correlation coefficient	NA	0.88***	0.90***	0.90***
Post-bronchodilator FEV_1_ (% predicted)				
Patients with data available, n	400	433	420	1253
Pearson’s correlation coefficient	−0.26***	−0.23***	−0.30***	−0.31***

*CAAT* Chronic Airways Assessment Test, *CAT* COPD Assessment Test, *COPD* chronic obstructive pulmonary disease, *EQ-5D-5L VAS* EuroQol 5-dimensions 5-level visual analogue scale, *FEV*_*1*_ forced expiratory volume in 1 s,* N* total number of patients in the sample, *n* number of patients in sample with available data, *NA* not applicable, *SGRQ* St George’s Respiratory Questionnaire

^a^Analyses were performed for patients with non-missing data; thus, number of observations differed for each variable

^b^To achieve an appropriate sample size, analysis of the CAT was performed in all NOVELTY patients who completed both the CAAT and CAT (N = 277). Correlation coefficients > 0.70 were regarded as strong; 0.4–0.7 moderate; and < 0.4 weak

^***^p < 0.0001

### Comparison between the CAAT and CAT

**Fig. 3:**
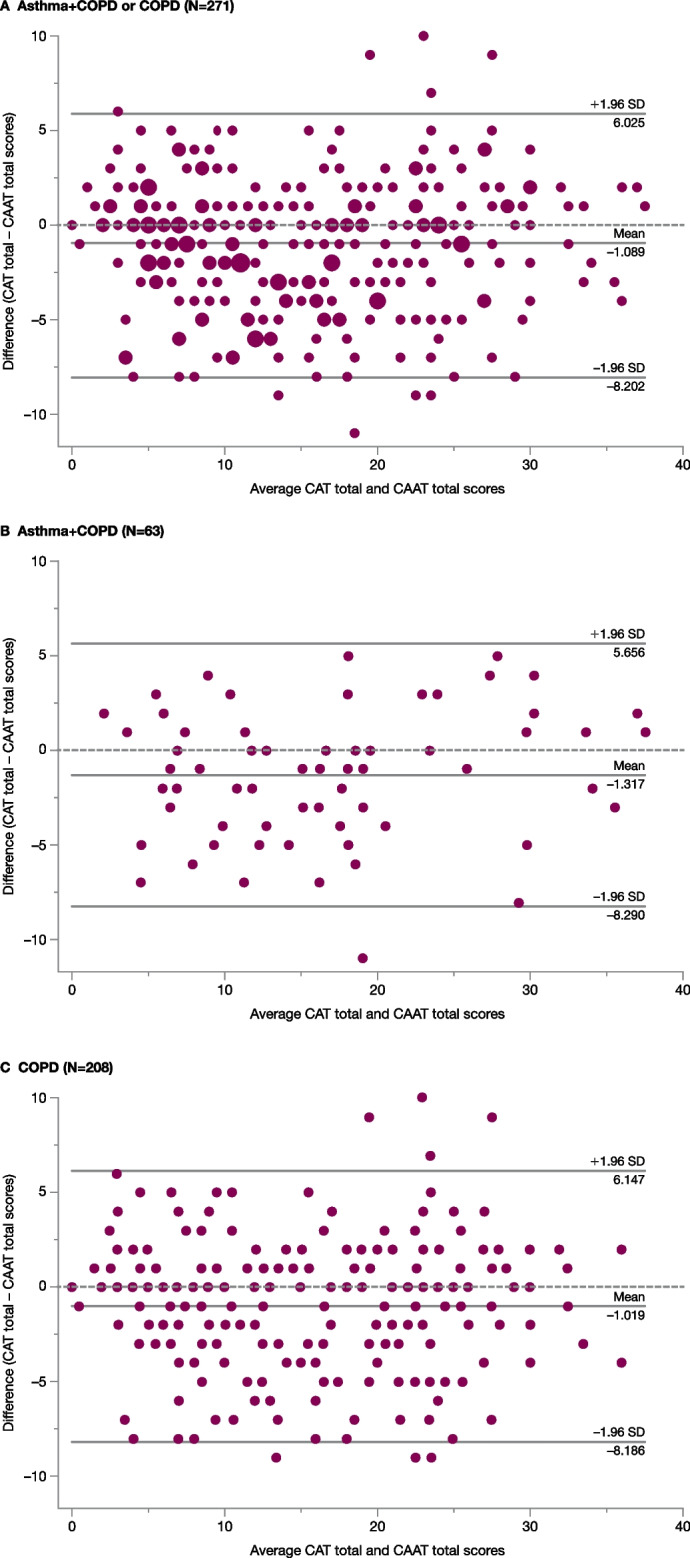
Bland–Altman plots of CAAT and CAT total scores in asthma + COPD, COPD, and both. Each small circle represents one patient, with the difference between CAAT and CAT total scores (CAT score – CAAT score) plotted against the average of the two scores. Jittering has been added to panel A for clarity where there were multiple superimposed circles. The central line shows the mean difference between the two measures, while the upper and lower lines show the limits of agreement (± 1.96 SD). Data for six patients who did not meet inclusion criteria have been excluded. *CAAT* Chronic Airways Assessment Test, *CAT* COPD Assessment Test, *COPD* chronic obstructive pulmonary disease, *N* total number of patients in the sample, *SD* standard deviation

Intraclass correlation indicated strong reliability between the CAAT and CAT (Additional file 1: Table S4). Bland–Altman plots showed no consistent difference between CAAT and CAT scores (Fig. 3).

## Discussion

These results show that the CAAT has strong psychometric properties and may be a suitable PRO for assessing health status in patients with asthma and/or COPD. While patients with asthma scored some items lower than patients with COPD in the DIF analysis, the overall differences in CAAT total score were small and their summed effect was below the CAT MCID and therefore unlikely to be of clinical importance. This suggests that CAAT scores in asthma and COPD are likely to reflect similar degrees of health impairment; however, this may not apply at an individual item level, where statistically and clinically significant differences were found, particularly for items 4 and 5 (breathlessness and limited home activity).

The observed emergence of CAAT item grouping into symptom-related and functional impact-related items suggests that a two-factor model would need to be explored further if CAAT domains were to be considered. The DIF analysis showed that this would only result in small improvements to precision, however. Like the CAT [13], the CAAT is designed to provide a single and easy-to-calculate measure of health status impairment, whereas a two-factor model would require a more complex scoring algorithm, introducing a barrier to its use. For these reasons, we have opted for a single total CAAT score as being suitable for the majority of CAAT applications.

The CAAT correlated strongly with the CAT, with no consistent difference seen in the Bland–Altman plots. As expected from previous studies of the CAT in COPD [13, 25], the CAAT consistently and strongly correlated with the SGRQ in all three diagnostic groups. It also correlated moderately with the EQ-5D-5L VAS, a generic measure of health status. However, as generally reported for other health status PROs [2, 8, 9], correlations between the CAAT and spirometry measures were weaker than those between the CAAT and other PROs, although patients with lower lung function tended to have worse CAAT scores as expected.

The 2022 GINA and GOLD reports emphasise the need for regular assessment of symptoms and their impact on patients with asthma and/or COPD [10, 14]; a need therefore exists for a clinically applicable PRO to use across asthma and COPD and in patients with both conditions. Recently, the Respiratory Symptoms Questionnaire was developed as a respiratory symptom tool for patients with asthma and/or COPD [26]; however, it is not designed to address the broader concept of health status. Our results suggest that the CAAT can assess health status in everyday clinical settings without adding undue patient burden. For instance, the CAAT was recently used in an investigation of the utility of patient-reported questionnaires in patients with or at risk of COPD [27].

Although the CAT was designed for use in patients with COPD, the CAAT demonstrated good performance in patients with physician-assigned asthma, asthma+COPD and COPD. Unlike some asthma and COPD questionnaires, the CAAT captures aspects of health status relevant to both asthma and COPD, including the impact on activity, sleep and energy level. Of the items included in the CAAT, only items 6 (confidence leaving home) and 8 (energy) are not already part of routine asthma assessment [10]. Although item 2 (phlegm) is not currently included in validated asthma symptom control tools, it is common in patients with asthma [28, 29]. Furthermore, previous qualitative patient interviews of asthma symptoms support the relevance of several CAAT items in patients with asthma [30]. The CAAT provides a single tool for standardised assessment of disease-specific health status in routine clinical practice across a range of obstructive lung diseases.

A key strength of this analysis is that it was performed using a range of measures within a large, real-world population of patients across primary and non-primary care settings.

Limitations of this analysis include the stratification of NOVELTY enrolment by physician-assessed severity (to ensure adequate and approximately equal sample sizes for subgroup analyses [12, 16]). Consequently, the patients in this analysis sample did not reflect a truly random sample of asthma and COPD populations in the community. Patients with asthma + COPD were relatively overrepresented in this analysis compared with the overall NOVELTY population (33% vs. 12%, respectively) [12], but this can give us some confidence in the reliability of our findings in these patients since it provided a large sample size. Finally, the sample size may have been overly conservative for some of the analyses, particularly the IRT, resulting in some analyses being slightly overpowered and detecting small but not clinically important differences.

Beyond the scope of this paper, future analyses should look in detail at the relationship between CAAT scores and a range of measures of severity relevant to asthma, asthma + COPD and COPD, and a longitudinal analysis to assess the performance of the CAAT over time. Further research is required to determine the CAAT score MCID, whether this differs from the CAT MCID [22], and investigate whether it applies across diagnostic and severity groups. Current asthma control instruments are poorly responsive in patients with severe asthma [31], so it will be important to determine the responsiveness of the CAAT in this patient group.

## Conclusion

This cross-sectional analysis is the first step in psychometrically evaluating the CAAT as a measure of health status in patients with asthma and/or COPD. It has demonstrated good cross-sectional psychometric properties and moderate-strong correlations with other health status measures, making it a suitable PRO instrument to assess the impact of obstructive lung disease in broad populations of patients with airways disease. Due to its brevity, the CAAT may be particularly relevant for routine clinical practice and ‘real-world’ effectiveness studies performed in patients in a routine care setting.

## Supplementary Information

**Additional file 1: Table S1** Patient demographics and clinical assessments by physician-assigned diagnosis and physician-assessed severity. **Table S2** Confirmatory factor analysis summary of invariance model testing fit statistics. **Table S3** Pearson’s correlations between CAAT score and additional spirometric assessments. **Table S4** Intraclass correlation coefficient data for CAT vs. CAAT items. **Fig S1** The Chronic Airways Assessment Test (CAAT), response options and scoring**. Fig S2** Summary of the number of patients enrolled in NOVELTY and patient samples randomly selected for psychometric analysis, according to physician-assigned diagnostic label. **Fig S3** Item response theory modelling of CAAT item information functions. **Fig S4** Linearity and dispersion in relationships between CAAT and SGRQ total scores at the individual patient level for patients with asthma and/or COPD in the total sample (**A**) and for patients with asthma (**B**), asthma + COPD (**C**) and COPD (**D**).

## Data Availability

Data underlying the findings described in this manuscript may be obtained in accordance with AstraZeneca’s data-sharing policy, described at https://astrazenecagrouptrials.pharmacm.com/ST/Submission/Disclosure.  Data for studies directly listed on Vivli can be requested through Vivli at https://vivli.org/. Data for studies not listed on Vivli could be requested through Vivli at https://vivli.org/members/enquiries-about-studies-not-listed-on-the-vivli-platform/. AstraZeneca Vivli member page is also available outlining further details: https://vivli.org/ourmember/astrazeneca/. The study protocol is available at https://astrazenecagrouptrials.pharmacm.com.

## Abbreviations

AIC: Akaike information criterion
AQ20: Airways Questionnaire 20
BIC: Bayesian information criterion
CAAT: Chronic Airways Assessment Test
CAT: COPD Assessment Test
CFI: Comparative fit index (Bentler)
CI: Confidence interval
COPD: Chronic obstructive pulmonary disease
df: Degrees of freedom
DIF: Differential item functioning
EFA: Exploratory factor analyses
EQ-5D-5L VAS: EuroQol 5-dimensions 5-level visual analogue scale
FEV_1_: Forced expiratory volume in 1 s
FVC: Forced vital capacity
ICC: Intraclass correlation coefficient
IRT: Item response theory
ML: Maximum likelihood
N: Total number of patients in the sample
n: Number of patients with non-missing data
NA: Not applicable
PRO: Patient-reported outcome
RMSEA: Root mean square error of approximation
SD: Standard deviation
SGRQ: St George’s Respiratory Questionnaire
SRMR: Standardised root mean residual

## References

[1] Monteagudo M, Rodríguez-Blanco T, Llagostera M, Valero C, Bayona X, Ferrer M, Miravitlles M (2013). Factors associated with changes in quality of life of COPD patients: a prospective study in primary care. Respir Med.

[2] Barley EA, Quirk FH, Jones PW (1998). Asthma health status measurement in clinical practice: validity of a new short and simple instrument. Respir Med.

[3] US Food and Drug Administration. Guidance for Industry. Patient-reported outcome measures: use in medical product development to support labeling claims. 2009. https://www.fda.gov/media/77832/download. Accessed 22 Apr 2022.

[4] US Food and Drug Administration. Chronic obstructive pulmonary disease: use of the St. George’s Respiratory Questionnaire as a PRO assessment tool. Guidance for industry. 2018. https://www.fda.gov/files/drugs/published/Chronic-Obstructive-Pulmonary-Disease--Use-of-the-St.-George%E2%80%99s-Respiratory-Questionnaire-as-a-PRO-Assessment-Tool-Guidance-for-Industry.pdf. Accessed 22 Apr 2022.

[5] European Medicines Agency. Reflection paper on the regulatory guidance for the use of health-related quality of life (HRQL) measures in the evaluation of medicinal products. 2005. https://www.ema.europa.eu/en/documents/scientific-guideline/reflection-paper-regulatory-guidance-use-healthrelated-quality-life-hrql-measures-evaluation_en.pdf. Accessed 22 Apr 2022.

[6] Wilson SR, Rand CS, Cabana MD, Foggs MB, Halterman JS, Olson L, Vollmer WM, Wright RJ, Taggart V (2012). Asthma outcomes: quality of life. J Allergy Clin Immunol.

[7] Cazzola M, MacNee W, Martinez FJ, Rabe KF, Franciosi LG, Barnes PJ, Brusasco V, Burge PS, Calverley PMA, Celli BR (2008). Outcomes for COPD pharmacological trials: from lung function to biomarkers. Eur Respir J.

[8] Jones PW, Quirk FH, Baveystock CM, Littlejohns P (1992). A self-complete measure of health status for chronic airflow limitation. The St. George's Respiratory Questionnaire. Am Rev Respir Dis.

[9] Hajiro T, Nishimura K, Jones PW, Tsukino M, Ikeda A, Koyama H, Izumi T (1999). A novel, short, and simple questionnaire to measure health-related quality of life in patients with chronic obstructive pulmonary disease. Am J Respir Crit Care Med.

[10] Global Initiative for Asthma. Global Strategy for Asthma Management and Prevention: 2022 Report. 2022. https://ginasthma.org/reports/. Accessed 30 Jun 2022.

[11] Cosío BG, Dacal D, Pérez de Llano L (2018). Asthma-COPD overlap: identification and optimal treatment. Ther Adv Respir Dis..

[12] Reddel HK, Vestbo J, Agustí A, Anderson GP, Bansal AT, Beasley R, Bel EH, Janson C, Make B, Pavord ID (2021). Heterogeneity within and between physician-diagnosed asthma and/or COPD: NOVELTY cohort. Eur Respir J.

[13] Jones PW, Harding G, Berry P, Wiklund I, Chen W-H, Kline LN (2009). Development and first validation of the COPD Assessment Test. Eur Respir J.

[14] Global Initiative for Chronic Obstructive Lung Disease. Global Strategy for the Diagnosis, Management, and Prevention of Chronic Obstructive Pulmonary Disease: 2022 Report. 2022. https://goldcopd.org/2022-gold-reports-2/. Accessed 22 Apr 2022.

[15] Karloh M, Fleig Mayer A, Maurici R, Pizzichini MMM, Jones PW, Pizzichini E (2016). The COPD Assessment Test: what do we know so far?: A systematic review and meta-analysis about clinical outcomes prediction and classification of patients into GOLD stages. Chest.

[16] Reddel HK, Gerhardsson de Verdier M, Agustí A, Anderson G, Beasley R, Bel EH, Janson C, Make B, Martin RJ, Pavord I (2019). Prospective observational study in patients with obstructive lung disease: NOVELTY design. ERJ Open Res..

[17] Chen WH, Lenderking W, Jin Y, Wyrwich KW, Gelhorn H, Revicki DA (2014). Is Rasch model analysis applicable in small sample size pilot studies for assessing item characteristics? An example using PROMIS pain behavior item bank data. Qual Life Res.

[18] Guilleux A, Blanchin M, Hardouin J-B, Sébille V (2014). Power and sample size determination in the Rasch model: evaluation of the robustness of a numerical method to non-normality of the latent trait. PLoS ONE.

[19] Streiner DL (2003). Starting at the beginning: an introduction to coefficient alpha and internal consistency. J Pers Assess.

[20] Samejima F (1969). Estimation of latent ability using a response pattern of graded scores. Psychometrika.

[21] Crane PK, Gibbons LE, Jolley L, van Belle G (2006). Differential item functioning analysis with ordinal logistic regression techniques: DIFdetect and difwithpar. Med Care.

[22] Kon SSC, Canavan JL, Jones SE, Nolan CM, Clark AL, Dickson MJ, Haselden BM, Polkey MI, Man WD-C (2014). Minimum clinically important difference for the COPD Assessment Test: a prospective analysis. Lancet Respir Med.

[23] Dancey CP, Reidy J (2017). Statistics without maths for psychology.

[24] Brown T, Jones T, Gove K, Barber C, Elliott S, Chauhan A, Howarth P (2018). Wessex Severe Asthma Cohort team. Randomised controlled trials in severe asthma: selection by phenotype or stereotype. Eur Respir J.

[25] Gupta N, Pinto LM, Morogan A, Bourbeau J (2014). The COPD assessment test: a systematic review. Eur Respir J.

[26] Karlsson N, Atkinson MJ, Müllerová H, Alacqua M, Keen C, Hughes R, Janson C, Make B, Price D, Reddel HK (2021). Validation of a diagnosis-agnostic symptom questionnaire for asthma and/or COPD. ERJ Open Res.

[27] Tamaki K, Sakihara E, Miyata H, Hirahara N, Kirichek O, Tawara R, Akiyama S, Katsumata M, Haruya M, Ishii T (2021). Utility of self-administered questionnaires for identifying individuals at risk of COPD in Japan: the OCEAN (Okinawa COPD casE finding AssessmeNt) study. Int J Chron Obstruct Pulmon Dis.

[28] Lange P, Parner J, Vestbo J, Schnohr P, Jensen G (1998). A 15-year follow-up study of ventilatory function in adults with asthma. N Engl J Med.

[29] Martínez-Rivera C, Crespo A, Pinedo-Sierra C, García-Rivero JL, Pallarés-Sanmartín A, Marina-Malanda N, Pascual-Erquicia S, Padilla A, Mayoralas-Alises S, Plaza V (2018). Mucus hypersecretion in asthma is associated with rhinosinusitis, polyps and exacerbations. Respir Med.

[30] Gater A, Nelsen L, Fleming S, Lundy JJ, Bonner N, Hall R, Marshall C, Staunton H, Krishnan JA, Stoloff S (2016). Assessing asthma symptoms in adolescents and adults: qualitative research supporting development of the asthma daily symptom diary. Value Health.

[31] Pavord ID, Korn S, Howarth P, Bleecker ER, Buhl R, Keene ON, Ortega H, Chanez P (2012). Mepolizumab for severe eosinophilic asthma (DREAM): a multicentre, double-blind, placebo-controlled trial. Lancet.

