# Supplementary material for: Chronic Airways Assessment Test: psychometric properties in patients with asthma and/or COPD
Source: Respir Res. 2023 Apr 8;24:106. doi: 10.1186/s12931-023-02394-6 (PMC10082977; doi:10.1186/s12931-023-02394-6)
Supplement: Supplementary file 1 — Additional file 1: Table S1 Patient demographics and clinical assessments by physician-assigned diagnosis and physician-assessed severity. Table S2 Confirmatory factor analysis summary of invariance model testing fit statistics. Table S3 Pearson’s correlations between CAAT score and additional spirometric assessments. Table S4 Intraclass correlation coefficient data for CAT vs. CAAT items. Fig S1 The Chronic Airways Assessment Test (CAAT), response options and scoring. Fig S2 Summary of the number of patients enrolled in NOVELTY and patient samples randomly selected for psychometric analysis, according to physician-assigned diagnostic label. Fig S3 Item response theory modelling of CAAT item information functions. Fig S4 Linearity and dispersion in relationships between CAAT and SGRQ total scores at the individual patient level for patients with asthma and/or COPD in the total sample (A) and for patients with asthma (B), asthma + COPD (C) and COPD (D). [file 12931_2023_2394_MOESM1_ESM.docx]

# Additional file 1: Supplementary materials

**Table S1** Patient demographics and clinical assessments by physician-assigned diagnosis and physician-assessed severity

| **Variable** | **Asthma (N=510)** | | | **Asthma+COPD^a^ (N=510)** | | | **COPD (N=510)** | | |
| --- | --- | --- | --- | --- | --- | --- | --- | --- | --- |
|  | **Mild (N=181)** | **Moderate (N=190)** | **Severe (N=139)** | **Mild (N=70)** | **Moderate (N=233)** | **Severe (N=207)** | **Mild (N=151)** | **Moderate (N=142)** | **Severe (N=217)** |
| Age, years, mean (SD) | 52.0 (16.5) | 55.3 (16.1) | 56.9 (13.4) | 63.8 (11.6) | 66.8 (9.1) | 63.9 (9.9) | 64.7 (10.7) | 67.9 (9.0) | 68.7 (8.8) |
| Female, n (%) | 121 (66.9) | 121 (63.7) | 86 (61.9) | 32 (45.7) | 108 (46.4) | 100 (48.3) | 58 (38.4) | 57 (40.1) | 88 (40.6) |
| Ethnicity, n (%) |  |  |  |  |  |  |  |  |  |
| African American | 5 (2.8) | 7 (3.7) | 3 (2.2) | 3 (4.3) | 6 (2.6) | 8 (3.9) | 7 (4.6) | 6 (4.2) | 2 (0.9) |
| Caucasian | 139 (76.8) | 142 (74.7) | 86 (61.9) | 60 (85.7) | 171 (73.4) | 167 (80.7) | 130 (86.1) | 120 (84.5) | 191 (88.0) |
| North-east Asian | 27 (14.9) | 28 (14.7) | 27 (19.4) | 7 (10.0) | 48 (20.6) | 21 (10.1) | 11 (7.3) | 15 (10.6) | 9 (4.1) |
| South-east Asian | 2 (1.1) | 3 (1.6) | 4 (2.9) | 0 | 3 (1.3) | 2 (1.0) | 0 | 0 | 1 (0.5) |
| Other | 8 (4.4) | 10 (5.3) | 19 (13.7) | 0 | 5 (2.1) | 9 (4.3) | 3 (2.0) | 1 (0.7) | 14 (6.5) |
| Time since diagnosis, years |  |  |  |  |  |  |  |  |  |
| Patients with data, n | 167 | 178 | 121 | 65 | 216 | 184 | 140 | 130 | 195 |
| Mean (SD) | 15.3 (15.2) | 18.9 (16.0) | 19.6 (17.1) | 21.3 (22.8) | 18.2 (18.8) | 24.3 (21.3) | 6.6 (9.1) | 8.1 (10.5) | 9.2 (7.9) |
| Post-bronchodilator FEV_1_ (% predicted) |  |  |  |  |  |  |  |  |  |
| Patients with data, n | 140 | 147 | 113 | 57 | 196 | 180 | 125 | 114 | 181 |
| Mean (SD) | 90.3 (16.9) | 88.0 (18.5) | 76.4 (23.9) | 82.5 (13.8) | 71.6 (17.9) | 56.4 (20.5) | 80.1 (19.9) | 65.1 (17.4) | 43.7 (16.5) |
| SGRQ total score^b^ |  |  |  |  |  |  |  |  |  |
| Patients with data, n | 175 | 188 | 137 | 68 | 229 | 205 | 146 | 139 | 216 |
| Mean (SD) | 23.4 (16.7) | 27.2 (19.3) | 40.8 (21.2) | 30.0 (20.0) | 35.2 (20.3) | 50.0 (21.6) | 31.6 (20.4) | 39.0 (20.7) | 50.3 (18.5) |
| EQ-5D-5L VAS score^b^ |  |  |  |  |  |  |  |  |  |
| Patients with data, n | 150 | 166 | 118 | 62 | 199 | 190 | 137 | 123 | 190 |
| Mean (SD) | 78.0 (14.4) | 76.0 (17.8) | 67.7 (18.9) | 71.1 (20.0) | 70.8 (18.1) | 62.1 (21.0) | 69.8 (18.5) | 67.9 (21.7) | 59.2 (19.1) |
| CAAT total score^c^ |  |  |  |  |  |  |  |  |  |
| Patients with data, n | 181 | 190 | 139 | 70 | 233 | 207 | 151 | 142 | 217 |
| Mean (SD) | 11.3 (7.1) | 12.9 (7.9) | 17.8 (8.5) | 14.0 (8.5) | 15.6 (8.1) | 20.0 (8.3) | 14.2 (8.1) | 15.7 (8.0) | 19.6 (7.5) |
| CAT total score^c^ |  |  |  |  |  |  |  |  |  |
| Patients with data, n | NA | NA | NA | 4 | 23 | 10 | 12 | 13 | 21 |
| Mean (SD) | NA | NA | NA | 9.8 (6.5) | 13.7 (8.1) | 20.4 (10.1) | 10.8 (9.0) | 14.2 (8.0) | 21.3 (7.8) |
| **All NOVELTY patients who completed both the CAAT and CAT (N=277)^d^** | | | | | | | | | |
| CAAT total score^c^ |  |  |  |  |  |  |  |  |  |
| Patients with data, n | NA | NA | NA | 10 | 34 | 22 | 59 | 76 | 76 |
| Mean (SD) | NA | NA | NA | 11.2 (4.9) | 14.4 (8.9) | 22.6 (9.2) | 12.2 (8.3) | 15.1 (7.8) | 19.9 (8.6) |
| CAT total score^c^ |  |  |  |  |  |  |  |  |  |
| Patients with data, n | NA | NA | NA | 10 | 34 | 22 | 59 | 76 | 76 |
| Mean (SD) | NA | NA | NA | 9.6 (4.7) | 13.4 (8.9) | 21.6 (10.1) | 11.4 (8.2) | 13.8 (7.9) | 19.1 (8.1) |

*CAAT* Chronic Airways Assessment Test, *CAT* COPD Assessment Test, *COPD* chronic obstructive pulmonary disease, *EQ-5D-5L VAS* EuroQol 5-dimensions 5-level visual analogue scale, *FEV_1_* forced expiratory volume in 1 second, *N* total number of patients in the sample, *n* number of patients with non-missing data, *NA* not applicable, *SD* standard deviation, *SGRQ* St George’s Respiratory Questionnaire
^a^For patients with asthma+COPD, severity was allocated as the higher of the two severity categories assigned by the physician for their asthma and their COPD; ^b^Range: 0–100; ^c^Range: 0–40; ^d^Data for all NOVELTY patients with asthma+COPD or COPD who completed the CAAT and CAT, including those not represented in the N=1530 total sample

**Table S2** Confirmatory factor analysis summary of invariance model testing fit statistics

| **Model** | **X^2^** | **df** | **p-value** | **CFI** | **RMSEA (90% CI)** | **SRMR** | **BIC** | **AIC** |
| --- | --- | --- | --- | --- | --- | --- | --- | --- |
| **Pooled total sample (N=1530)** |  |  |  |  |  |  |  |  |
| No modifications | 643.8 | 20 | 0 | 0.87 | 0.14 (0.13–0.15) | 0.06 | NA | NA |
| With modifications^a^ | 76.1 | 13 | 0 | 0.99 | 0.06 (0.04–0.07) | 0.02 | NA | NA |
| **Asthma vs. COPD (N=1020)** |  |  |  |  |  |  |  |  |
| Structural invariance^b^ with modifications,  ML estimation | 234.8 | 32 | 0 | 0.93 | 0.11 (0.10–0.13) | 0.12 | 26678.0 | 26402.1 |
| Measurement invariance^c^ with modifications, ML estimation | 166.6 | 32 | 0 | 0.96 | 0.09 (0.08–0.11) | 0.08 | 26609.8 | 26333.8 |
| **Asthma vs. Asthma+COPD (N=1020)** |  |  |  |  |  |  |  |  |
| Structural invariance^b^ with modifications,  ML estimation | 201.3 | 32 | 0 | 0.95 | 0.10 (0.09–0.12) | 0.11 | 26546.5 | 26270.6 |
| Measurement invariance^c^ with modifications, ML estimation | 137.2 | 32 | 0 | 0.97 | 0.08 (0.07–0.09) | 0.07 | 26482.4 | 26206.5 |
| **COPD vs. Asthma+COPD (N=1020)** |  |  |  |  |  |  |  |  |
| Structural invariance^b^ with modifications,  ML estimation | 328.3 | 32 | 0 | 0.90 | 0.14  (0.12–0.15) | 0.16 | 27099.2 | 26823.3 |
| Measurement invariance^c^ with modifications, ML estimation | 94.1 | 32 | 0 | 0.98 | 0.06  (0.08–0.15) | 0.03 | 26865.0 | 26589.1 |

*AIC* Akaike information criterion, *BIC* Bayesian information criterion, *CAAT* Chronic Airways Assessment Test, *CFI* comparative fit index (Bentler), *CI*confidence interval, *COPD* chronic obstructive pulmonary disease, *df* degrees of freedom, *ML* maximum likelihood, *N* total number of patients in the sample, *NA* not applicable, *RMSEA* root mean square error of approximation, *SRMR* standardised root mean residual
^a^Modifications required to optimise fit by accounting for correlated residuals associated with item types (items 1–3 vs. 4–8)_._ Modifications to the CAAT structural models were permissible and expected to account for covariance patterns due to item and rating scale design; ^b^Fixed factor loadings; ^c^Fixed factor loadings and means

**Table S3** Pearson’s correlations between CAAT score and additional spirometric assessments

| **Variable^a^** | **Asthma (N=510)** | **Asthma+COPD (N=510)** | **COPD (N=510)** | **Total sample (N=1530)** |
| --- | --- | --- | --- | --- |
| Post-bronchodilator FVC (% predicted) |  |  |  |  |
| Patients with data available, n | 400 | 432 | 419 | 1251 |
| Pearson’s correlation coefficient | −0.24*** | −0.27*** | −0.29*** | −0.29*** |
| Post-bronchodilator FEV_1_/FVC (% predicted) |  |  |  |  |
| Patients with data available, n | 400 | 431 | 419 | 1250 |
| Pearson’s correlation coefficient | −0.12* | −0.03 | −0.19** | −0.18*** |

*CAAT* Chronic Airways Assessment Test, *COPD* chronic obstructive pulmonary disease, *FEV_1_* forced expiratory volume in 1 second, *FVC* forced vital capacity, *N* total number of patients in the sample, *n* number of patients in sample with available data
^a^Analyses were performed for patients with non-missing data; thus, number of observations differed for each variable
*p<0.05; **p<0.001; ***p<0.0001

**Table S4** Intraclass correlation coefficient data for CAT vs. CAAT items in all NOVELTY patients who completed both

|  | **ICC** | **F** | **95% CI** | **Sigma s** | **Sigma r** | **Sigma e** |
| --- | --- | --- | --- | --- | --- | --- |
| **CAT vs. CAAT (total sample; N=271,^a^ df=270)** |  |  |  |  |  |  |
| Item 1: Cough | 0.750 | 7.0 | (0.693–0.798) | 1.240 | 0 | 0.716 |
| Item 2: Phlegm | 0.825 | 10.4 | (0.783–0.860) | 1.392 | 0 | 0.642 |
| Item 3: Chest tightness | 0.762 | 7.6 | (0.705–0.809) | 1.308 | 0.123 | 0.721 |
| Item 4: Breathlessness | 0.817 | 10.0 | (0.773–0.853) | 1.484 | 0.066 | 0.700 |
| Item 5: Home activity | 0.839 | 11.8 | (0.797–0.873) | 1.480 | 0.122 | 0.636 |
| Item 6: Confidence | 0.793 | 9.2 | (0.734–0.839) | 1.325 | 0.168 | 0.655 |
| Item 7: Sleep | 0.762 | 7.6 | (0.705–0.809) | 1.365 | 0.131 | 0.751 |
| Item 8: Energy | 0.839 | 11.4 | (0.800–0.871) | 1.407 | 0.028 | 0.616 |
| Total | 0.914 | 24.1 | (0.881–0.936) | 8.539 | 0.754 | 2.515 |
| **CAT vs. CAAT (Asthma+COPD subgroup; N=63, df=62)** |  |  |  |  |  |  |
| Total | 0.927 | 29.7 | (0.871–0.958) | 9.339 | 0.878 | 2.465 |
| **CAT vs. CAAT (COPD subgroup; N=208, df=207)** |  |  |  |  |  |  |
| Total | 0.909 | 22.5 | (0.874–0.933) | 8.298 | 0.699 | 2.534 |

*CAAT* Chronic Airways Assessment Test, *CAT* COPD Assessment Test, *CI* confidence interval, *COPD* chronic obstructive pulmonary disease, *df* degrees of freedom, *ICC* intraclass correlation coefficient, *N* number of patients in the sample with non-missing data
^a^Data for six patients who did not meet inclusion criteria have been excluded

**Fig S1** Summary of the number of patients enrolled in NOVELTY and patient samples randomly selected for psychometric analysis, according to physician-assigned diagnostic label


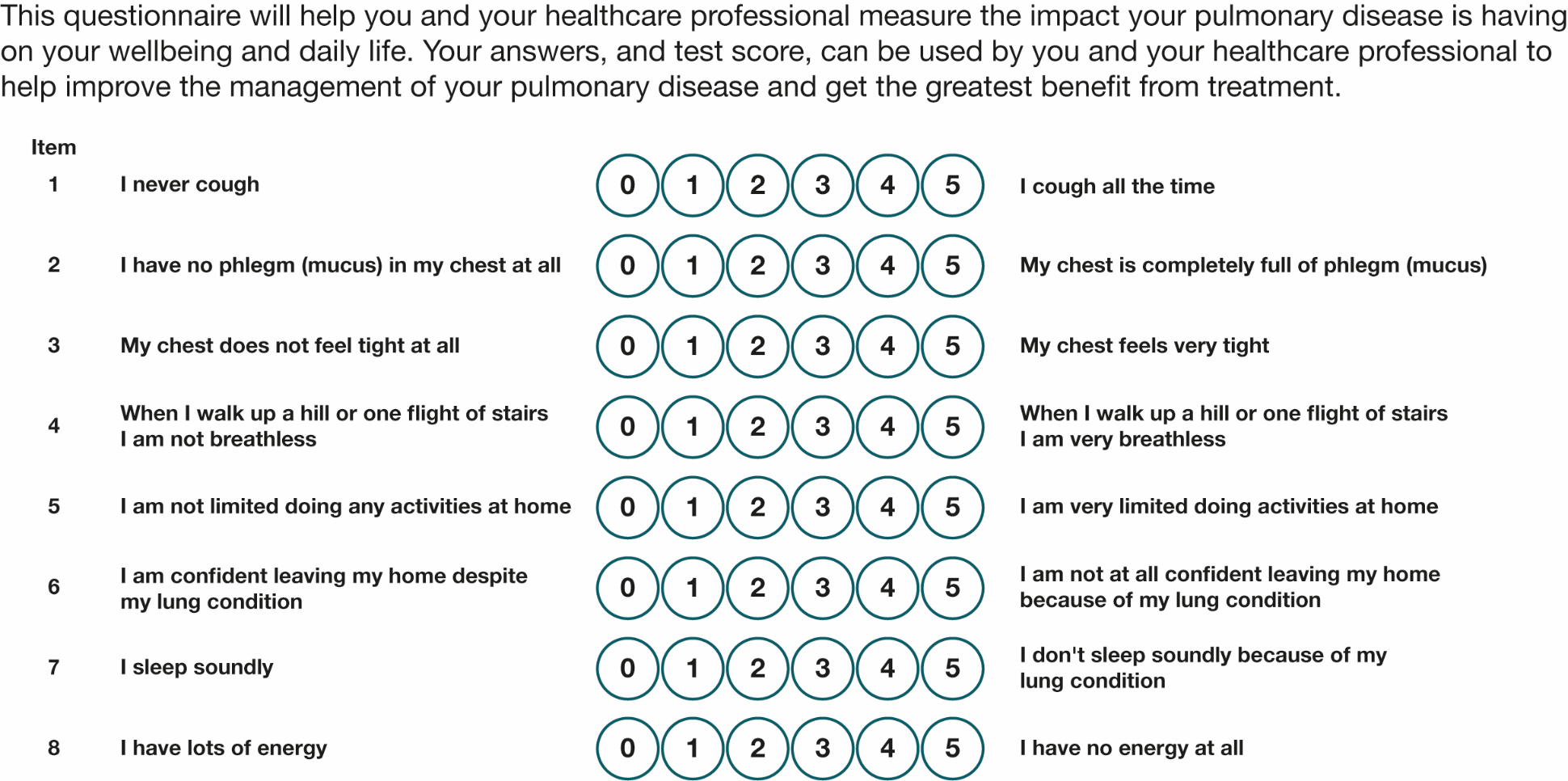


The COPD Assessment Test is the copyright of GlaxoSmithKline; the CAAT will similarly be placed under copyright
*CAAT* Chronic Airways Assessment Test, *COPD* chronic obstructive pulmonary disease

**Fig S2** Summary of the number of patients enrolled in NOVELTY and patient samples randomly selected for psychometric analysis, according to physician-assigned diagnostic label


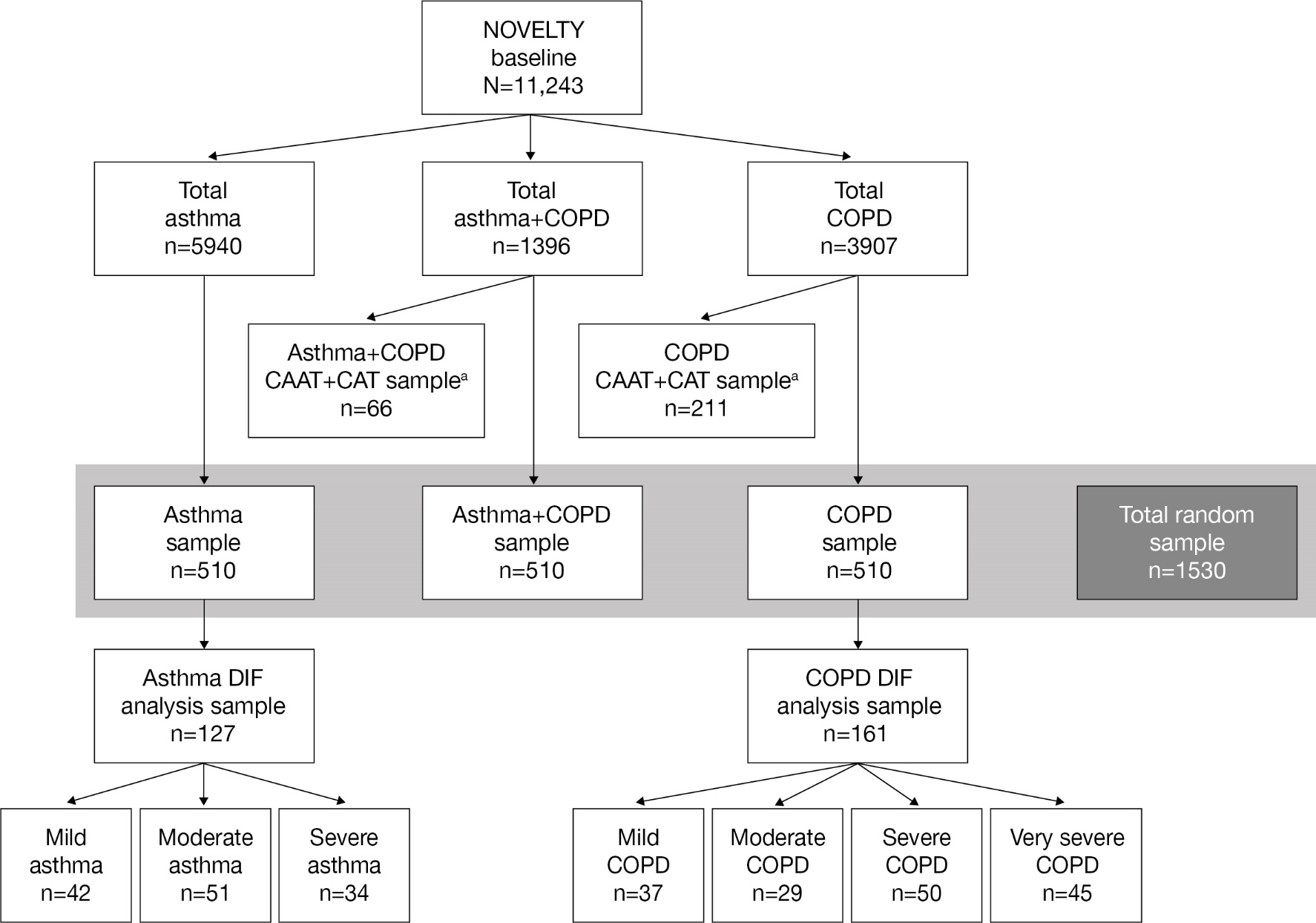


The DIF sample size was larger for COPD than for asthma to reflect an additional severity scoring category of ‘very severe’ for COPD. No patients with asthma were rated by clinicians as having ‘very severe’ disease.

*CAAT* Chronic Airways Assessment Test, *CAT* COPD Assessment Test, *COPD* chronic obstructive pulmonary disease, *N* total number of patients in the NOVELTY population, *n* number of patients in the diagnostic group or analysis sample, *NOVELTY* NOVEL observational longiTudinal studY
^a^CAAT + CAT samples include all patients in the asthma+COPD and COPD groups who completed both the CAAT and CAT; these patients were also included in the overall asthma+COPD and COPD samples

**Fig S3** Item response theory modelling of CAAT item information functions


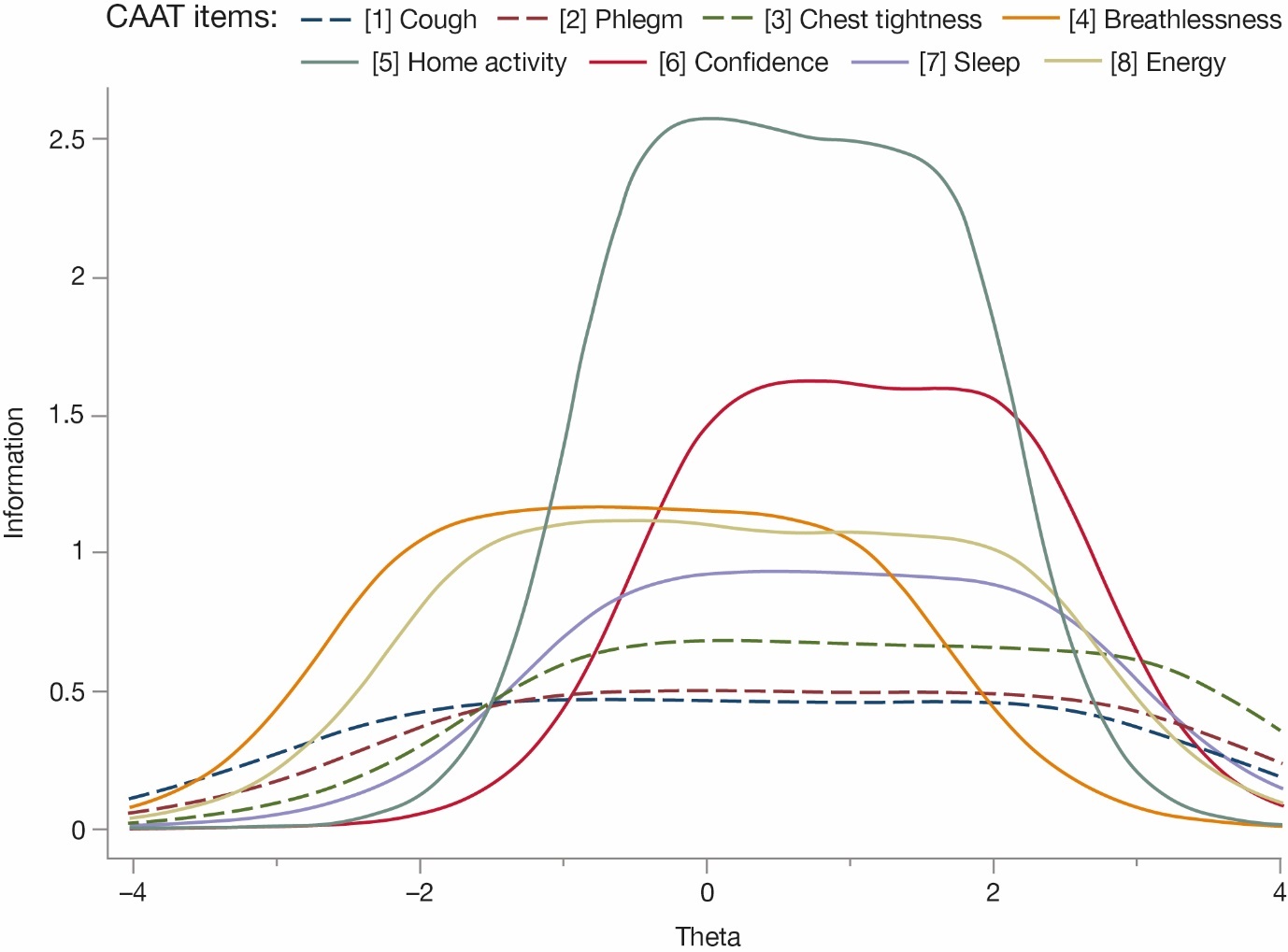


A higher level of information implies more precision [1]. Theta represents the continuum of patient responses to CAAT health status items. Items relating to symptoms (items 1–3) had broad coverage across theta but were less informative; items relating to functional impact (items 4–8) provided more information but had a narrower range
*CAAT* Chronic Airways Assessment Test

**Fig S4** Linearity and dispersion in relationships between CAAT and SGRQ total scores at the individual patient level for patients with asthma and/or COPD in the total sample (**A**) and for patients with asthma (**B**), asthma+COPD (**C**) and COPD (**D**)

**
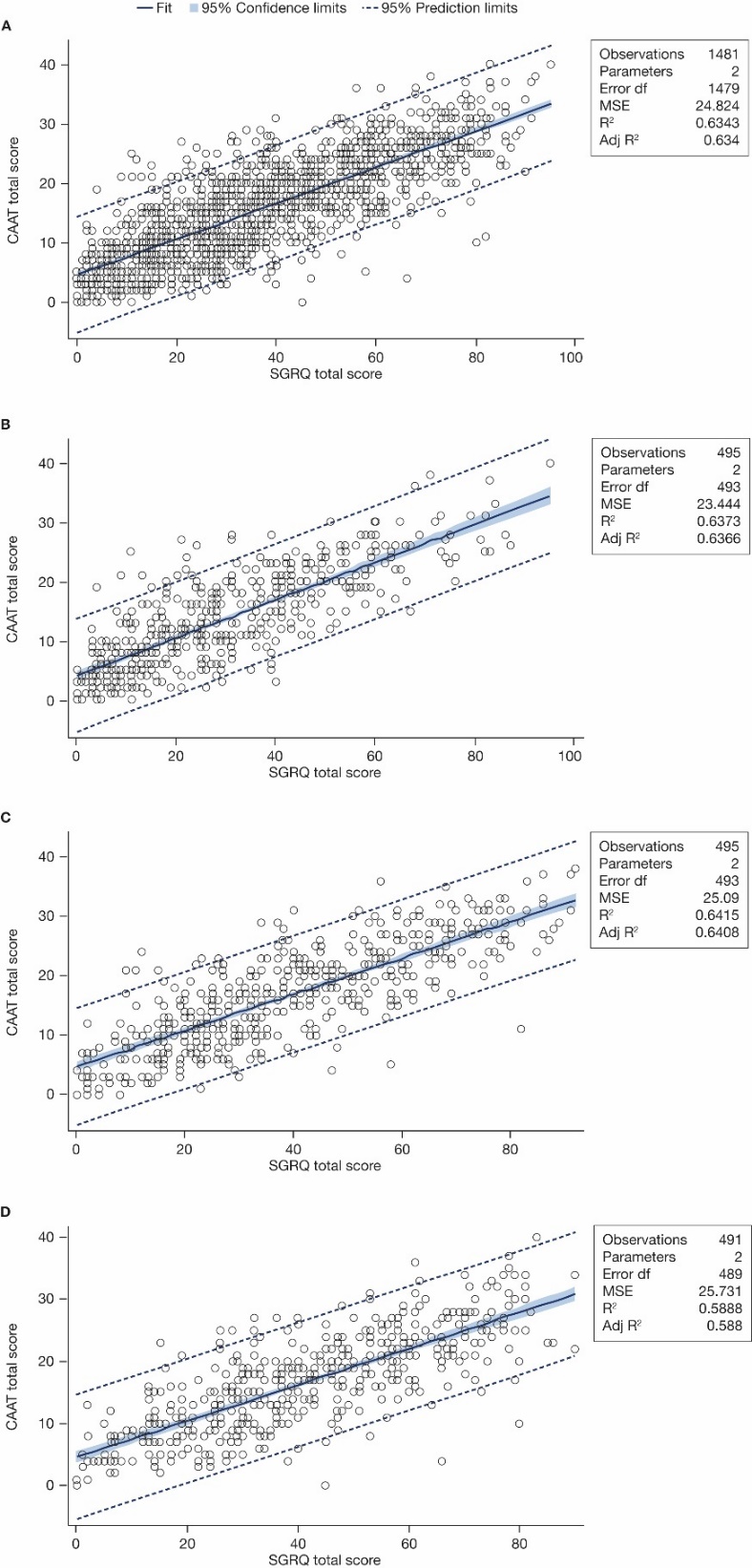
**

*CAAT* Chronic Airways Assessment Test, *df* degrees of freedom, *MSE* mean squared error, *SGRQ* St George’s Respiratory Questionnaire

# References

1. Cappelleri JC, Lundy JJ, Hays RD. Overview of classical test theory and item response theory for the quantitative assessment of items in developing patient-reported outcomes measures. Clin Ther. 2014;36:648-662.
